# The NDR family of kinases: essential regulators of aging

**DOI:** 10.3389/fnmol.2024.1371086

**Published:** 2024-05-13

**Authors:** Kevin Jonischkies, Miguel del Angel, Yunus Emre Demiray, Allison Loaiza Zambrano, Oliver Stork

**Affiliations:** ^1^Department of Genetics and Molecular Neurobiology, Institute of Biology, Otto-von-Guericke University Magdeburg, Magdeburg, Germany; ^2^Center for Behavioral Brain Science, Magdeburg, Germany; ^3^Center for Intervention and Research on Adaptive and Maladaptive Brain Circuits Underlying Mental Health (C-I-R-C), Jena-Magdeburg-Halle, Germany; ^4^German Center for Mental Health (DZPG), Jena-Magdeburg-Halle, Germany

**Keywords:** nuclear Dbf2-related (NDR) kinases, aging, cellular senescence, autophagy, brain aging, neuroinflammation, nutrient sensing and signaling, DNA repair

## Abstract

Aging is defined as a progressive decline of cognitive and physiological functions over lifetime. Since the definition of the nine hallmarks of aging in 2013 by López-Otin, numerous studies have attempted to identify the main regulators and contributors in the aging process. One interesting group of proteins whose participation has been implicated in several aging hallmarks are the nuclear DBF2-related (NDR) family of serine-threonine AGC kinases. They are one of the core components of the Hippo signaling pathway and include NDR1, NDR2, LATS1 and LATS2 in mammals, along with its highly conserved metazoan orthologs; Trc in *Drosophila melanogaster*, SAX-1 in *Caenorhabditis elegans*, CBK1, DBF20 in *Saccharomyces cerevisiae* and orb6 in *Saccharomyces pombe*. These kinases have been independently linked to the regulation of widely diverse cellular processes disrupted during aging such as the cell cycle progression, transcription, intercellular communication, nutrient homeostasis, autophagy, apoptosis, and stem cell differentiation. However, a comprehensive overview of the state-of-the-art knowledge regarding the post-translational modifications of and by NDR kinases in aging has not been conducted. In this review, we summarize the current understanding of the NDR family of kinases, focusing on their relevance to various aging hallmarks, and emphasize the growing body of evidence that suggests NDR kinases are essential regulators of aging across species.

## Introduction

Aging is broadly characterized as the time-dependent decline in organism fitness, leading to an elevated risk of intrinsic mortality associated with diseases such as coronary heart disease, cancer, or neurodegenerative diseases, among many others. Based on the growing body of aging research, nine hallmarks of aging were proposed in 2013 regarding the molecular and cellular aspects underlying aging mechanisms. Each hallmark inherently manifests in physiological aging, and their experimental impairment accelerates the aging process, while experimental amelioration delays aging and increases lifespan. These criteria collectively define and highlight the key features that contribute to the complex phenomenon of aging (López-Otín et al., 2013).

The original nine aging hallmarks included Cellular senescence, deregulated nutrient signaling, loss of proteostasis, mitochondrial dysfunction genomic instability, epigenetic alterations, altered intracellular communication, telomere attrition and stem cell exhaustion. After a decade of intense aging research, three additional hallmarks were proposed: chronic inflammation, impairment in macroautophagy and dysbiosis (López-Otín et al., 2023). It is important to emphasize that the hallmarks of aging do not exist as discrete biological entities; instead, they form a network of interconnected processes that frequently interact with each other. This interconnectedness is largely attributed to central signaling pathways that overlap across the aging hallmarks. Consequently, there is a significant interest in comprehending such pathways and identifying novel regulators that could serve as potential targets for anti-aging interventions.

The Nuclear Dbf2-related kinases (NDR) are part of the NDR/LATS (large-tumor-suppressor) subfamily of AGC (protein kinase A/G/C PKA/PKG/PKC-like). They are evolutionarily conserved from plants to mammals, and they were first described as core components of the Hippo signaling pathway (Hergovich, 2016). While four NDR-kinases are known to exist in vertebrates, four homologs have been described in invertebrates and more than 10 kinases are present in fungi, yeasts and plants (Table 1). As part of the Hippo pathway, NDR Kinases have an important function in the regulation of growth and organ size across tissues and species (Ma et al., 2019). In mammals, NDR1/2 and LATS1/2, together with their upstream activators mammalian sterile 20-like kinases (MST) 1 and 2 form the core cascade of the Hippo pathway (Chan et al., 2005; Hergovich et al., 2006; Praskova et al., 2008; Du et al., 2015; Tang et al., 2015; Gundogdu and Hergovich, 2016; Liu et al., 2016; Kurz et al., 2018). Activated LATS1/2 and NDR1/2 together with their co-activator MPS1-binder-related (MOB)-1 can directly phosphorylate the downstream transcription factors Yes-associated-protein (YAP) and WW domain–containing transcription regulator protein 1 (TAZ), leading to their cytosolic retention and degradation (Zhao et al., 2007, 2010; Liu et al., 2010). When unphosphorylated, YAP/TAZ shuttles to the nucleus and binds to the transcriptionally enhanced associate domains (TEAD) causing transcription of its downstream genes. Similarly, in *D. melanogaster*, the NDR1/2 homolog Tricornered (Trc) and LATS1/2 homolog Warts (Wts), are activated by the MST1/2-homolog Hippo and cause downstream phosphorylation and proteasomal degradation of the YAP homolog Yorkie (Yki) (Staley and Irvine, 2012).

**Table 1:** List of known and predicted NDR kinases.

**Protein**	**Gene**	**Taxonomy**
NDR1	*STK38/Stk38*	Mammals
NDR2	*STK38L/Stk38l*	Mammals
LATS1	*LATS1/Lats1*	Mammals
LATS2	*LATS2/Lats2*	Mammals
Trc	*trc*	*D. melanogaster*
Warts	*wts*	*D. melanogaster*
SAX-1	*sax-1*	*C. elegans*
WARTS	*wts-1*	*C. elegans*
CBK1	*CBK1*	*S. cerevisiae*
DBF20	*DBF20*	*S. cerevisiae*
DBF2	*DBF2*	*S. cerevisiae*
orb6	*orb6*	*Schizosaccharomyces pombe*
sid2	*sid2*	*Schizosaccharomyces pombe*
COT1	*cot-1*	*Neurospora crassa*
^*^CpCot1	*cot1*	*Claviceps purpurea*
^*^TB3	*TB3*	*Colletotrichum trifolii*
^*^TBPK50	Unknown	*Trypanosoma brucei*
Ukc1	*ukc1*	*Ustilago maydis*

^*^Predicted by homology.

NDR1, NDR2, LATS1, and LATS2 are the main mammalian NDR kinases. NDR1/2 is the ortholog of Trc and SAX1, and together they are the homologs of the LATS family of proteins that also includes Warts/WARTS. The evolutionary relationship with the other NDR kinases is still under debate, but it is accepted that CBK1, orb6, Ukc1, COT1 and their protein orthologs constitute the sister group of NDR1/2, SAX-1 and Warts/WARTS, and that DBF2 and DBF20 are the most distantly related kinases (Tamaskovic et al., 2003).

The roles of NDR kinases have been extensively characterized across various species, implicating them in the regulation of diverse cellular processes. These kinases play key roles in controlling size, migration, cell cycle, inflammation, cell signaling, proteostasis, transcription, trafficking, apoptosis, and, more recently, have emerged as critical components in neuronal differentiation, plasticity, synaptogenesis, and cognition (Zallen et al., 2000; Stork et al., 2004; Demiray et al., 2018; Léger et al., 2018; Madencioglu Kul, 2019; Madencioglu et al., 2021), underscoring their role as important regulators of neuronal biology. On the other hand, numerous studies show how NDR kinases can play a maladaptive role in disease, particularly in cancer and inflammation and evidence points out that they might contribute to neurodegeneration as well (Tacutu et al., 2011). While the precise role of NDR kinases in aging remains to be fully understood, there is ample evidence in the literature that strongly suggests their significance in the biology of aging. Furthermore, despite the increasing research linking NDR kinases to essential processes crucial for survival, and in the brain, that relate to cognition and memory, studies investigating the role of NDR kinases on biological fitness and longevity are still lacking.

## Cellular senescence and chronic inflammation

Perhaps the most widely characterized process, and one of the earliest pieces of evidence that linked NDR kinase function to aging, relates to inflammation. In the first iteration of the Hallmarks of aging, increased inflammation was considered a feature of altered intercellular communication (López-Otín et al., 2013). A more updated understanding of the inflammatory processes that occur during aging “inflammageing” makes it clear that altered communication by increased inflammation is not the only contributor to aging, but rather that there exists chronic inflammation which is the result of many intersecting pathways that integrate altered signals from other hallmarks processes like loss of proteostasis, genomic instability, mitochondrial dysfunction, and others, but particularly from cellular senescence (López-Otín et al., 2023). Mechanistically, the literature suggests that the way in which NDR kinases participate in inflammation overlaps with the regulation of inflammation through the Senescence-associated-secretory phenotype (SASP), hence, in this review, we address these two hallmarks in the same chapter.

Cellular senescence was first described as a replicative limit that most eukaryotic cell types can acquire irreversibly. Further research thoroughly demonstrated that senescence is a response to several signals, often related to stress, such as the shortening of the telomeres and accumulation of DNA damage after many replication cycles (Funayama and Ishikawa, 2007). Although the phenotype varies from cell to cell, some commonalities can be found among senescent cells. One of the main characteristics is that cells undergo a permanent cell cycle arrest mainly in the G_1_/S phase progression, though there is plenty of evidence demonstrating that senescence can also permanently arrest the cells in the G_2_/M transition (Chien et al., 2011). Overall, it has been widely demonstrated that prolonged arrest by activation of cell cycle regulators leads to cellular senescence (Alcorta et al., 1996; Stein et al., 1999; Shtutman et al., 2017; Safwan-Zaiter et al., 2022), supporting the idea that pathways that play a role in the regulation of the cell cycle are central to the regulation of aging and lifespan (Dottermusch et al., 2016; Seim et al., 2016; Campos et al., 2018). The other main characteristic is that although replication stops, senescent cells are highly active and acquire a SASP that includes secretion of mainly, but not exclusively, proinflammatory molecules, chemokines proteases and growth factors (Coppé et al., 2010). Cellular senescence, as well as other hallmarks of aging, display antagonistic pleiotropic features, it is considered to have evolved as a mechanism to assist in wound healing, tissue remodeling, and the prevention of malignant cellular transformation; senescent cells accumulate and evade clearance by the immune system through a not-yet-understood mechanism in aging organisms. Consequently, via the SASP, these cells become the primary contributors to chronic tissue inflammation and damage. Even though the regulation of the cell cycle progression and cellular senescence are intrinsically connected, it has been demonstrated that post-mitotic cells such as neurons can also become senescent and contribute to disease progression (Musi et al., 2018; Dehkordi et al., 2021; Herdy et al., 2022). A final but very important feature of senescent cells is that they are highly resistant to apoptosis, which is achieved partially by the upregulation of anti-apoptotic proteins such as B-cell lymphoma 2 (BCL-2) or B-cell-lymphoma-extra large (BCL-xL) (Hu et al., 2022).

### NDR kinases regulate the cell cycle progression

The mammalian NDR kinases are known regulators of the cell cycle, which implicates them directly in a key aspect of cellular senescence. *In vitro*, both NDR1 and NDR2 interact with the CyclinD1/CDK4 complex, which drives cell cycle progression. Mechanistically, CyclinD1 has been shown to increase the kinase activity of NDR1/2 by reducing its autoinhibition and promoting the G_1_/S cell cycle progression (Du et al., 2013). It is known that in the canonical Hippo pathway, NDR1/2 is activated by phosphorylation in the Thr444/Thr442-residue by the MST1/2 kinases. Moreover, *in vitro* work shows that NDR1/2 can also become phosphorylated by MST3 exclusively in the G_1_ phase. This phosphorylation controls the NDR1/2 kinase activity on p21 during the cell cycle, as it was demonstrated that depletion of NDR1/2 increased p21 stability and induced G_1_ arrest (Cornils et al., 2011).

The other mammalian NDR kinases, LATS1 and LATS2, have also been found to participate in the cell cycle transition. It was shown that, independently of its kinase activity, LATS1/2 can inhibit human cancer cell proliferation and induce a G_2_/M arrest. The mechanism by which this occurs is yet not completely understood, but research suggests that LATS1 and LATS2 act independently in distinct pathways in cell cycle progression. LATS1 binds to Cell division control protein 2 (CDC2) and inhibits the kinase activity of both Cyclin A/CDK1 and Cyclin B/CDK1 protein complexes. On the other hand, LATS2 decreases the kinase activity of the Cyclin B/CDK1 and the Cyclin E/CDK2 complex exclusively. The consequence is that overexpression of LATS1/2 can prevent the G_2_/M transition (Yang et al., 2001) and act as a negative regulator of the cell cycle progression. A finding that could seem contradictory was later revealed in a model of replicative senescence of human fibroblasts, which shows an increase in AMP-activated protein kinase 5 activity, and the subsequent phosphorylation of LATS1 at the S464, decreases LATS1 stability. Accordingly, inhibition of LATS1 activity accelerated replicative senescence (Humbert et al., 2010). This suggests that there are several pathways in which LATS1 might exert their role in the regulation of the cell cycle. A strong indication of this comes from the fact that overexpression of AMPK5 did not affect p53 activity, thus it is likely that the p53-p21 axis is not involved in this pathway. Arguably p16, a selective inhibitor of CDK4 and CDK6 kinases, is the most well-characterized CDK inhibitor that participates in the cell cycle arrest of senescent cells, mainly in the G_1_/S transition. This is achieved by maintaining the CDK4/6 downstream target, the retinoblastoma protein (pRB) in a hypophosphorylated state (Safwan-Zaiter et al., 2022).

Even in a mouse neuronal cell line, p16 expression has been associated with resistance to apoptosis when cell cycle regulators like Cyclin D1 are overexpressed in terminally differentiated cells (Kranenburg et al., 1996). Besides the inactivation of the pRB, it has been shown that in human fibroblasts, p16 activation leads to an increase of reactive oxygen species (ROS) mediated by a positive feedback loop that involves the downstream activation of the protein kinase C delta and downregulation of LATS1, in a mechanism that irreversibly blocks the cell cycle progression (Takahashi et al., 2006). In this same direction, it was reported that LATS2 participates in the repression of E2F genes in cellular senescence after expression of pRB, particularly from the dimerization partner, RB-like, E2F and multi-vulval class B (DREAM)-repressor complex, and the formation of the senescence-associated histone foci (SAHF) and the increased beta-galactosidase activity, characteristic of senescent cells. Remarkably, it was also found that both *RB*1 and *LATS1* loci are mapped in close proximity to the 13q locus, which is often lost in tumourigenic cell lines and can impair the induction of cell cycle arrest (Tschöp et al., 2011). Finally, another key piece of evidence that links NDR kinases in the permanent arrest of the cell cycle in senescent cells comes from a series of papers published by Aylon et al., in which they use multiple murine and human cell lines to investigate and describe a pathway that involves the translocation of LATS2 from the centrosomes to the nucleus in the context of mitotic stress, where it binds to Mouse double minute 2 homolog (MDM2), thus increasing p53 stability and transcription of downstream targets such as *CDKN1A* (p21) and *LATS2*, creating a positive feedback loop that helps to maintain the cell cycle arrest (Aylon et al., 2006). Moreover, they showed that LATS2 deficiency allows cells to escape oncogene-induced senescence (OIS) by HRAS (Aylon et al., 2009). The malignant transformation of cells is often linked to intrinsic causes, such as mutations in the DNA, and among them, mutations in *RAS* genes are one of the most aggressive and main risk factors of cancer. Interestingly, the expression of KRAS is sufficient to increase the NDR1 protein levels, and inhibition of NDR1 induces apoptosis and also increases p21 and LATS2 levels, possibly as a compensatory mechanism.

These data together suggest that although NDR kinases might have distinct roles within the pathways, there is a wide overlap of functions. It has also been shown that *NDR2* mRNA levels are increased in pancreatic cancer patients which allows for malignancy of transformed cells (Grant et al., 2017). Although this observation is contradictory to the role of NDR kinases in inducing cell cycle arrest in cellular senescence, it suggests that in cells that escape the cell cycle arrest, NDR kinases might allow for survival and an increase in the malignity of the tumors by hyperactivation of growth and facilitating migration possibly through MOB-mediated pathways and Hippo signaling. For example, it was shown that human MOB2 competes with MOB1 for binding to NDR1/2 as a negative regulator (Kohler et al., 2010) in a model of DNA damage and that independently of NDR1/2, MOB2 promotes survival and the cell cycle progression in human cells through inhibition of p21 and p53 (Gomez et al., 2015). Supporting this idea, it was shown that, unlike MOB1 or MOB2, MOB3 inhibits LATS1/2 signaling within the Hippo pathway and allows for continued proliferation of cells even after OIS (Dutchak et al., 2022), potentially contributing to cancer progression. This is consistent with the observation that LATS2 enforces cell cycle arrest during OIS by engaging with pRB and promoting the silencing of E2F genes (Tschöp et al., 2011). In summary, it is evident that NDR kinases play a significant role in cell cycle regulation through various mechanisms across different species. While their direct involvement in the permanent cell cycle regulation of cellular senescence has not been directly addressed, we suggest that they could be crucial components of the machinery enforcing the cell cycle arrest in senescent cells.

### NDR kinases might facilitate resistance to apoptosis in senescent cells

Many studies have examined the complementary and yet contradictory relationship between cellular senescence and apoptosis mechanism during aging. The current consensus in the field is that both mechanisms have evolved as a regulatory process for tissue modeling and to prevent the malignant transformation of cells (Campisi, 2013), thus many of the molecular mechanisms that induce apoptosis, cellular senescence or apoptosis resistance in senescent cells overlap and are governed by the same proteins such as p53, BCL-2 or extracellular-signal-regulated kinases (ERK) signaling (Childs et al., 2014). It has been hypothesized that one main factor that determines whether the cell will enter senescence or apoptosis relates to cellular stress levels. Extreme cellular stress resulting from various factors such as irreversible DNA damage, exposure to UV irradiation, oncogenic activation, or withdrawal of growth factors triggers the intrinsic apoptotic pathway (Visser and Yang, 2010). It has been shown that both human LATS1 and LATS2 participate in parallel but independent apoptosis mechanisms, one in which LATS1 engages p53 independently of BAX which increases caspase 3 activity (Yang et al., 2001), and the other in which LATS2 overexpression downregulates BCL-2 and BCL-xL, resulting in a cascade that increases caspase 9 processing (Ke et al., 2004).

Other additional pathways in which LATS1/2 has been shown to be involved in the intrinsic apoptotic pathway are the upregulation of LATS2 by Checkpoint kinase 1 (CHEK1) upon activation of the DNA Damage Response (DDR) (Aylon et al., 2009), and the activation of the protease OMI/HTRA2 by LATS1, which is required for caspase cascade activation upon mitochondrial permeabilization (Kuninaka et al., 2005). Except for the case of excitotoxicity, aging neurons are highly resistant to stress and apoptosis (Kole et al., 2013), probably as a consequence of some of them becoming senescent (Si et al., 2021). Activating Transcription Factor 4 (ATF4) plays an important role in neuronal apoptosis and it is known that it induces the transcription of the C/EBP homologous protein, which activates the protein p53 Upregulated Modulator of Apoptosis (PUMA) in mouse cortical neurons (Galehdar et al., 2010). Additionally, it was shown both in mouse and human cell lines that EI2F-α promotes the translation of ATF4 and subsequent stabilization of LATS1 which increases cell death after oxidative stress (Rajesh et al., 2016). A downregulation on this pathway has yet to be demonstrated as a possible link between NDR kinases and the resistance of apoptosis in aging neurons.

### NDR kinases might participate in the regulation of inflammatory cytokines of the SASP

SASP is a senescence state characterized by the secretion of a diverse array of molecules, ranging from pro-inflammatory cytokines to growth factors. While research on neuronal SASP is limited, compelling *in vitro* evidence from rat primary neurons suggests that senescent neurons also undergo SASP, a process driven by the transcription factor GATA Binding Protein 4 (GATA4) and impaired autophagic flux (Moreno-Blas et al., 2019). The potential involvement of NDR kinases in the regulation and secretion of SASP remains largely unexplored. However, NDR kinases have been associated with the regulation of established SASP components, such as Tumor necrosis factor-alpha (TNF-α) which has been implicated in the heightened neuroinflammatory response and cognitive impairment during aging (Habbas et al., 2015; Probert, 2015). It has also been shown that murine NDR1 positively regulates the production of TNF-α and Interleukin 6 (IL-6) by directly binding to Smad ubiquitination regulatory factor 1 (SMURF 1) which leads to ubiquitination and degradation of mitogen-activated protein kinase 2 (MEKK2) and reducing the transcription of its downstream targets (Wen et al., 2015). Besides NDR2, another study reported that NDR1 activity also increases TNF-α dependent activation of the transcription factor nuclear factor “kappa-light-chain-enhancer” of activated B-cells (NF-κB) in various human tumor cell lines (Shi et al., 2012), supporting the important role of NDR1/2 in the regulation of TNF-α activity. Interestingly, Braitsch et al. (2019) found an opposing role for LATS1/2 in NF-κB regulation and reported that *LATS1/2* KO increases NFκB activation and *vanin1* expression, which favors epithelial-mesenchymal transition. NFκB is one of the main transcription factors that accumulate in the chromatin of senescent cells and controls the expression of SASP genes (Chien et al., 2011; Freund et al., 2011), suggesting an important link between NDR kinases and the control of the SASP.

In summary, NDR kinases serve as crucial regulators of the cell cycle. While not directly addressed, the existing literature strongly points out their involvement in enforcing cell cycle arrest in senescent cells. When this effect is circumvented, the expression of NDR kinases can have deleterious consequences, aiding cells in survival and migration, particularly through their role in the Hippo pathway. Additionally, it is plausible that NDR kinases participate in the pathways dictating whether a cell enters senescence or undergoes apoptosis and eventually providing senescent cells with resistance to apoptosis. A maladaptive feature of this role is that increased NDR expression is often observed in transformed cells due to its pro-survival effect, facilitating tumor progression and malignancy. Finally, it is well-known that mechanistically, NDR kinases can participate in the increased inflammatory activity during aging, and possibly in the control of SASP. As documented in the previous literature, functions of NDR1/2 and LATS1/2 have been associated with contradictory roles, which is compounded by a general lack of insight into their essential role as regulators of aging, a point that is highlighted throughout this review.

## Nutrient signaling

Some of the most evolutionarily conserved pathways across species are associated with how cells perceive the presence or absence of nutrients and regulate intracellular metabolism. These pathways collectively have profound connections with the regulation of lifespan and are notably recognized for their extensive crosstalk among the hallmarks of aging. Among these, four major pathways have been extensively investigated: the Insulin and Insulin growth factor 1 signaling (IIS) pathway, the mammalian target of rapamycin (mTOR) pathway, the AMPK pathway (Stallone et al., 2019), and, notably in the brain, the sirtuin (SIRT) signaling. Importantly, NDR kinases emerge as a significant upstream regulator of nutrient sensing mechanisms, given the growing body of research that demonstrated their significant roles across all four major pathways. Moreover, there is also evidence in the literature which shows that NDR kinases interact with known anti-aging proteins that also exercise their function within the nutrient signaling pathways like Klotho and SIRT1. In the following chapter, the known molecular interactions of NDR within the four nutrient-sensing pathways are summarized.

### NDR kinases participate in AMPK-signaling

There is enough evidence in the literature that links NDR kinases to AMPK signaling, which is required for the proper function of AMPK signaling, one of the main pathways that cells have to respond to nutrient deprivation. Moreover, NDR activity within the pathway has also been shown to play a maladaptive role, particularly by promoting age-related diseases such as metabolic syndrome. Besides nutrient sensing, activation of the AMPK pathway with metformin prolongs lifespan across species (Novelle et al., 2016), counteracts the development of neurodegenerative diseases (Rotermund et al., 2018) and in middle-aged mice improves cognitive performance by increasing autophagy of the hippocampus (Kodali et al., 2021). Additionally, clinical studies suggest that metformin treatment inhibits memory loss in diabetic adults (Ng et al., 2014). *In vitro*, evidence shows that the activation of the AMPK pathway by nutrient starvation, metformin treatment, or its more potent analog phenformin increases the phosphorylation and degradation of the NDR kinase downstream target YAP, either by direct phosphorylation of YAP on its Ser-94 residue and thus disruption of its interactions with TEAD or indirectly through increase of LATS1/2 activity (DeRan et al., 2014; Mo et al., 2015). In this line, it was also reported that silencing of LATS1/2 is sufficient to ablate the effect of energy stressors on AMPK-dependent YAP phosphorylation (DeRan et al., 2014). Interestingly LATS1/2 is not always essential for the activation of YAP via AMPK signaling since it was shown that AMPK can inhibit the oncogenic transformation of LATS-null mouse embryonic fibroblasts by directly inhibiting YAP activity (Mo et al., 2015).

Similarly, in *Drosophila melanogaster* larval central brain and ventral nerve cord, it has been shown that AMPK together with the AMPK upstream regulator liver kinase B1 (Lkb1), can inactivate Yki independent of the NDR Kinases (Gailite et al., 2015). AMPK signaling also seems to play a role in the shuttling of human YAP. A signaling cascade that involves AMPK, LK1B and LATS1/2, promotes the interaction of YAP with the WNT signaling protein Disheveled, and its eventual nuclear export (Lee et al., 2018). The mechanism involves the activation of AMPK, the downstream increase of SCRIB (Liu et al., 2020), Angiomiotin-like protein 1 and 2 (AMOTL1/2) and Angiomotin (AMOT) (DeRan et al., 2014) protein levels, which are involved in LATS1/2 stabilization and therefore AMPK-dependent YAP degradation. Furthermore, another study showed that Klotho^±^ mice, a widely used knockout mouse model in aging research that displays extremely short life span and premature aging (Kuro-o et al., 1997) display a weaker AMPK-LATS1 interaction as well as decreased AMPK-dependent phosphorylation of YAP (Luo et al., 2023). Although the exact mechanism has not been described yet, these data suggest that there is a direct interaction between AMPK and LATS1 which might be facilitated by the anti-aging protein Klotho.

The other mammalian NDR kinases have also been linked to AMPK signaling. Overexpression of NDR1 in mice decreases AMPK and the downstream Acetyl-CoA-carboxylase phosphorylation, which leads to enhanced *de novo* lipogenesis and increased incidence of non-alcoholic fatty liver diseases (NAFLD). In accordance, NDR1 overexpression alone is sufficient to cause NAFLD and increase inflammation in the liver under a regular diet. Interestingly, NDR1 exhibits a deleterious effect on the disease. A liver-specific KO of *Stk38* ameliorated high-fat-died (HFD) induced insulin resistance, hepatic inflammation, and lipid accumulation, as well as can reduce the cholesterol and triacylglycerol (TAG) levels (Rawat et al., 2023), which are major indicators of metabolic syndrome. Moreover, besides a systemic effect, NAFLD has been linked to reduced cognitive functions in adults (Bertolotti, 2014; Seo et al., 2016; Takahashi et al., 2017; Weinstein et al., 2018, 2019) and in mice, HFD has been shown to impair amygdala and hippocampus-dependent memory consolidation and cause neuroinflammation during aging (Spencer et al., 2017). Additionally changes in cholesterol as well as TAG levels have been linked to the neuronal aging process and the etiology of various age-related diseases including Alzheimer's (AD), Parkinson's (PD) and Huntington's disease (Spitler and Davies, 2020; Nunes et al., 2022). It has been extensively demonstrated that the responsiveness of rodent AMPK to nutrient deprivation decreases during aging (Salminen and Kaarniranta, 2012). Based on previous studies demonstrating the significant interactions of AMPK and NDR signaling, we suggest that the age-related reduction of AMPK signaling might involve a decreased downstream inhibition of YAP by NDR-kinases and increased lipid accumulation and higher incidence of NAFLD observed during aging.

### NDR kinases display extensive crosstalk with mTOR signaling

The complex intercommunication between mTOR signaling and NDR kinases has been intensively studied both in Drosophila and in mammals. The first study that linked NDR-Kinases to mTOR showed that *Drosophila* salivary gland-specific KO of *wts* leads to decreased cell death, caspase activity and autophagy, while the expression of a dominant negative form of *Drosophila* Tor, abolished the effect of the *wts* KO (Dutta and Baehrecke, 2008). Furthermore, a substantial body of evidence indicates a two-way communication between NDR kinases and TOR signaling, with particular relevance for neuronal function.

In *Drosophila* Trc phosphorylation at T449 was shown to be dependent on TorC2 in class IV sensory neurons and required for the regulation of dendritic tilting (Koike-Kumagai et al., 2009). Along those lines, it is known that semaphorins promote neuronal substance adhesion in *Drosophila* by blocking dendrite crossing in a signaling cascade that involves the semaphorin receptor Sema-2B, TorC2, the β-integrin-subunit myospheroid (Mys), and Trc (Meltzer et al., 2016). Additionally, *trc* KO displays increased synaptic boutons number in the neuromuscular junction (NMJ), and a decrease in Yki phosphorylation therefore increasing Yki-dependent transcription of the Wiskott-Aldrich Syndrome Protein (Wasp) (Natarajan et al., 2015). It is well-known that Wasp regulates the synapse development in the neuromuscular juntion (Coyle et al., 2004; Khuong et al., 2010; Nahm et al., 2010)through actin polymerization (Stradal et al., 2004), hence a model was proposed where Trc acts downstream of TorC2 and regulates Wasp levels and modulates actin polymerization and synapse formation (Natarajan et al., 2015). In that regard, an accumulation of filamentous actin (F-actin) in the *Drosophila* brain has recently been suggested to occur during aging and disruption of actin polymerization in aged animals rescues autophagy levels, restores the youthful neuronal cell phenotype, and slows brain aging (Schmid et al., 2023). Besides their role as essential regulators of development in mammals (Kramer et al., 2022), the *Drosophila* Wasp homolog has been implicated in the increase of neuronal F-actin during aging in the brain, which suggests that their activity could be related to the pathological loss of proteostasis and deregulated nutrient sensing.

Another interesting role of NDR kinases in mTOR signaling is that NDR1 has been shown to increase mTOR-Complex 1 (mTORC1) activity and Rabin8 phosphorylation, leading to autophagy inhibition (Amagai et al., 2015), while on the other hand, LATS2 participates in the suppression of mTORC1 activity (Gan et al., 2020), which is implicated in pancreatic β-cell apoptosis and autophagic cell death under diabetic conditions. Additionally, the same study found that *Lats2* KO rescues high fat-died induced phosphorylation of the ribosomal S6 protein and p62 accumulation, which stems from increased mTORC1 signaling and impaired autophagic flux, respectively. Besides mediating autophagy-dependent effects under diabetic conditions, LATS2 is also colocalized with autophagosomes and accumulates upon treatment with bafilomycin or chloroquine, which are commonly used inhibitors of lysosomal function, suggesting that LATS2 is degraded by the autophagosome-lysosomal machinery (Yuan et al., 2021). Although not fully characterized, these data suggest an evolutionary conserved regulatory axis of NDR kinases by Tor/mTOR that participates in neuronal function and disease.

### NDR kinases in insulin signaling

The IIS is one of the major nutritional signaling pathways and has been thoroughly implicated in lifespan control, aging and age-related pathologies across several species (Altintas et al., 2016; Mathew et al., 2017; Zia et al., 2021). The canonical activation of IIS commences with the binding of either insulin or IGF1 to the insulin receptor, leading to autophosphorylation of its cytoplasmic tyrosine residues. Subsequently, adapter proteins such as Insulin-receptor-substrates (IRS) bind to the phosphorylated residues, initiating the activation of the Phosphoinositide 3-kinases (PI3K), Protein kinases B (PKB/AKT, hereafter referred to as AKT), and mTOR cascade which results in the downregulation of forkhead box O (FOXO) transcription factors. This constitutes one of the best-defined regulatory networks central to the control of lifespan and longevity (Wang et al., 2014; Webb and Brunet, 2014; Klotz et al., 2015; Martins et al., 2016). The IIS has been shown to play a neuroprotective role during mammalian brain aging, and low levels of IGF1 are linked to several age-related diseases (Zia et al., 2021).

Within this regulatory axis, the activation of AKT has been demonstrated to reduce MST1 and LATS1 activity. Similarly, inhibition of the PI3K-AKT pathway leads to an increase in MST1 and LATS1 phosphorylation that enforces cytosolic localization and degradation of YAP. Intriguingly, in a non-phosphorylated state, YAP remains in the nucleus and downregulates the phosphatase and tensin homolog (PTEN), a negative regulator of the PI3K-AKT pathway. This intricate interplay forms a positive feedback loop that illustrates the complex and nuanced regulation within this signaling network (Qian et al., 2021). AKT activation and its downstream effect on NDR-Kinases seem to be dependent on the DNA-double-strand-break-repairing protein DNA-dependent protein kinase, catalytic subunit (DNA-PKcs), as suggested by experiments in human glioblastoma cell lines. Inhibition of DNA-PKcs decreases AKT phosphorylation at its S473 residue and NDR1 activation upon glucose deprivation. Additionally, AKT and DNA-PKcs-dependent activation of MST1 in these conditions increases phosphorylation of NDR1 at S281 and T282 (Shiga et al., 2020). Supporting the role of NDR kinases in enforcing a positive feedback loop on AKT signaling, adenoviral overexpression of NDR1 in mice was shown to reduce the activation of AKT by phosphorylation at S473 and T308. This resulted in impairment in glucose-dependent Insulin signaling and increased inflammation, demonstrated by higher levels of Interleukin-6 and TNF- α (Rawat et al., 2023).

Even though it has not been demonstrated yet, this could mean that an increase or impairment in the function of NDR kinases during aging might have a maladaptive role which promotes the age-related loss of cognitive function through an increase in ISS.

### NDR2 kinases and Sirtuins

Sirtuins are nicotinamide adenine dinucleotide (NAD^+^) dependent protein deacetylases that have been thoroughly implicated in the regulation of aging and lifespan. It has been demonstrated in models ranging from *C. elegans* to humans that SIRT1 function and protein levels decrease with age and that overexpressing or reconstituting SIRT1 function can increase lifespan and delay aging (Satoh et al., 2013; Kilic et al., 2015). Moreover, the age-related alteration of autophagy has been linked to a decrease in SIRT1 (Xu et al., 2020), and the other way around, SIRT1 plays an essential role in the regulation of mammalian autophagy through the regulation of several key steps of the autophagic pathway (Kitada et al., 2016). In the brain, SIRT1 is predominantly expressed in neurons within the hippocampus and plays a crucial role in memory and plasticity (Michán et al., 2010). In human cell lines, it was shown that the acetyltransferases p300 and CREB-Binding Protein (CBP) can specifically acetylate NDR2 at K463, while SIRT1 is the major deacetylate of NDR2 (Tang and Yu, 2019). A similar study showed that LATS1 is also under the regulation of Sirtuins. Like NDR2, LATS1 can be acetylated by p300, but deacetylation takes place by distinct Sirtuins, namely SIRT3 and SIRT4 (Yang S. et al., 2022). Further, SIRT7 deacetylates the DNA damage-binding protein 1 (DDB1), which under acetylated conditions is involved in ubiquitination and degradation of LATS1 (Mo et al., 2017). While the evidence linking NDR kinases to Sirtuins is still limited, it underscores an entirely novel regulatory mechanism of NDR kinases that positions them within the central regulatory network of lifespan and longevity.

### Other functions of NDR-kinases in nutrient signaling

Lastly, in human tumor cells with high glucose uptake, the O-GlcNAc transferase (OGT) has been shown to O-GlcNacylate YAP and disrupt its interaction with LATS, decreasing YAP-phosphorylation and degradation, therefore increasing YAP-dependent transcription in an AMPK-independent manner. Interestingly OGT is under YAP-dependent transcription, forming another feedback loop with the involvement of NDR-kinases in nutrient signaling pathways (Peng et al., 2017).

One consequence of the increased metabolic activity of senescent cells is elevated glycogenesis through a mechanism that involves the activation of glycogen synthase, downregulation of Glycogen synthase kinase 3 beta (GSK-3β) and an increase in reactive oxygen species (ROS) (Seo et al., 2008). It has been shown that GSK-3 β signaling is altered also in the murine brain during aging, particularly in the hippocampus (Drulis-Fajdasz et al., 2018). Interestingly, *in vitro* it has been shown that GSK-3β also inhibits NDR1 activation, emphasizing the protective role of NDR1 in preventing cell death under increased oxidative stress (Enomoto et al., 2012). Similarly, in human melanoma cells, LATS1 has also been implicated in the regulation of ROS, and *LATS1* knockdown results in increased oxidative stress (Kazimierczak et al., 2021).

## Loss of proteostasis and disabled macroautophagy

A balance between protein synthesis and degradation is fundamental for the cell's functional integrity, a concept encapsulated in the term “proteostasis”. Proteostasis represents the intricate regulatory mechanisms that ensure the proper handling of proteins within a cellular environment. This equilibrium involves the coordinated orchestration of protein synthesis by the ribosomal machinery, folding and transport assisted by chaperones, and eventual degradation mainly by the proteasome and the lysosomal pathway (Mizushima et al., 2008; Hartl et al., 2011; Koga et al., 2011). Maintaining proteostasis is essential for cellular health, and disruptions in this delicate balance are at the core of several diseases such as myopathy, metabolic disorders, cardiovascular disease, ataxia, cataracts or persistent nephrotic syndrome. The importance of proteostasis becomes even more evident during aging and in neurodegenerative disorders, like AD, PD or amyotrophic lateral sclerosis (ALS) which are characterized by the accumulation of misfolded proteins (Labbadia and Morimoto, 2015). Autophagy involves a series of networks that ensure the delivery and degradation of biomolecules and organelles through the lysosome and is one of the main mechanisms that controls proteostasis which is prone to be altered during aging. Macroautophagy is the most well-characterized form of autophagy and since the original paper by López-Otín et al. (2023) postulated its decline as an aging hallmark a decade ago, disruption in macroautophagy has drawn great attention as a main contributor to aging and disease, that now it is considered to be a hallmark of its own, given that macroautophagy participates not only in proteolysis, but also in cell-to-cell communication, antigen presentation, cell growth, nutrient sensing, and many more (Nieto-Torres, 2021; Münz, 2022; Piletic et al., 2023). Here we summarize the role of NDR kinases both in the regulation of autophagy and proteostasis, highlighting how their maladaptive function during aging promotes disease, leaving the involvement in other features related to autophagy for the following chapters.

### NDR kinases positively regulate autophagy

NDR kinases seem to play a major role in the regulation of (macro) autophagy. Some early evidence pointed out that a loss-of-function mutation of Wts impaired autophagy and contributed to tissue degeneration. Interestingly, overexpression of the downstream effectors of Wts, Yki and scalloped (Sd), failed to rescue the effect, and that the activation of PI3K-Akt-Tor-pathway was essential for the phenotype in mutant flies (Dutta and Baehrecke, 2008), which indicated that Wts participated in autophagy regulation independently of the core Hippo signaling, through Tor signaling. Wts also plays a crucial role in autophagy-mediated cholesterol trafficking and subsequent steroid production in *Drosophila*. Yki-dependent transcription of the miRNA bantam enhances Tor activation and inhibits ecdysone receptor signaling, a potent inducer of autophagy in *Drosophila*. This process results in diminished mobilization and trafficking of cholesterol (Texada et al., 2019). In mammals, NDR1/2 enhances the inhibitory impact of the guanine nucleotide-exchange factor Rabin8 on autophagy. This effect was shown to be independent of Rabin8′s guanine-exchange-factor activity toward its downstream target RAB8 or others like SEC15 and Mammalian TRAPPII-specific subunit 130 (mTRS130) (Amagai et al., 2015). In the same study, it was observed that the silencing of NDR1/2 leads to a reduction in mTORC1 activity, but silencing Rabin8 did not have an impact on it. These observations suggest that besides Rabin8, additional mechanisms through which NDR1/2 inhibits autophagy exist, for instance by activating mTORC1 (Coyle et al., 2004; Joffre et al., 2015).

More direct recent evidence supporting the pleiotropic role of NDR kinases in the regulation of autophagy is that NDR1 interacts with BECLIN1 and other proteins that are part of the same complex required for early autophagosome formation, and silencing NDR1 in human cell lines or its homolog Trc in *Drosophila* impaired autophagy (Joffre et al., 2015). Interestingly, NDR1 has been demonstrated to phosphorylate the nuclear exit protein exportin1 (XPO1) at S1055, thereby influencing the nuclear exit of BECLIN1, YAP and itself (Martin et al., 2019). This discovery showcases the idea that the NDR family of kinases have multiple and sometimes opposing roles, in this case, in the regulation of autophagy. A different mechanism has been proposed for the role of NDR2 in autophagy. Under conditions of nutrient starvation, the E3-ubiquitin ligase Tripartite Motif Containing 27 (TRIM27) ubiquitinates NDR2 at L6 and L11 which enhances NDR2 activity, resulting in the downstream phosphorylation of Unc-51-like kinase 1 (ULK1) at S495. Notably, this ULK1 phosphorylation leads to increased binding of ULK1 to TRIM27 and ULK1 polyubiquitination. Consequently, this polyubiquitination promotes the enhanced proteasomal turnover of ULK1, suggesting an inhibitory role for NDR2 in autophagy initiation. Intriguingly, the frequent presence of TRIM27 overexpression in breast cancer (BC) patients is associated with tumorigenesis, potentially through the inhibition of ULK1-mediated autophagy (Yang Y. et al., 2022).

Due to its high level of similitude, it is usually assumed that NDR1 and NDR2 have complementary and sometimes overlapping functions. A notable exception is in the CNS, as it has been suggested that NDR1 expression decreases postnatally and that NDR2 is the main functional kinase of them in the adult rodent brain (Zallen et al., 2000). Recently, a double KO of *Stk38* and *Stk38l* in mice has been linked to neurodegeneration in both adult and prenatal mice. This neurodegeneration is associated with impaired autophagy and the mechanism appears to involve the absence of NDR1/2-dependent phosphorylation of the endocytosis protein RAPH1. This deficiency leads to impaired endocytosis of the autophagy protein Autophagy-related protein (ATG) 9A at the presynapse, subsequently resulting in reduced axonal trafficking (Roşianu et al., 2023). Lastly, one notable interaction of NDR kinases and autophagy is that of the *Drosophila* Trc with Atg8 (Tsapras et al., 2022), which is the homolog of the Microtubule-associated proteins 1A/1B light chain 3 (LC3A/B). Although this interaction has not been described in mammals so far, it indicates that the NDR kinases are tightly associated with the regulation of autophagy and have to be considered one of the main regulators of macroautophagy. With respect to other types of autophagy, it is known that NDR1 is needed for mitochondrial clearance through mitophagy upon Extracellular matrix (ECM) detachment of Ras-transformed cells. NDR1 also participates in chaperone-assisted selective autophagy (CASA), a type of chaperone-mediated autophagy (Carra et al., 2008; Gamerdinger et al., 2009; Arndt et al., 2010; Klimek et al., 2017), by binding and inhibiting the function of the cochaperone BCL-2-associated athanogene 3 (BAG3) (Klimek et al., 2019) in tension-dependent degradation of filamins (Arndt et al., 2005, 2010). It is important to note that BAG3-mediated protein clearance is critical for the disposal of proteins associated with neurodegeneration like the AD-related protein TAU (Ji et al., 2019), Huntingtin (Klimek et al., 2017) and ALS-related Superoxide dismutase 1 (SOD1) (Crippa et al., 2010; Gamerdinger et al., 2011). This function of NDR kinases is already being explored for the treatment of age-related diseases. For instance, activating LATS1 in mice, either through the use of the traditional Chinese medicine compound Paris saponin VII or Long non-coding RNAs (lncRNAs) like RP1-59D14.5, can effectively reduce the growth of BC or prostate cancer cells in mice. This reduction is attributed to the induction of autophagy in breast and prostate cancer cells, respectively (Xiang et al., 2022; Zhong et al., 2022). Finally, the extract of the plant *Radix scrophulariae* has recently been shown to inhibit thyroid growth in a rat-hyperthyroidism model, through MST-LATS1-dependent autophagy activation (Zhang et al., 2023).

### NDR kinases participate in correct protein folding

NDR kinases also seem to participate in protein folding by interacting with the chaperone HSP (Heat shock protein) 90, which is one of the two main chaperones involved in maintaining proteostasis through the regulation of protein folding and stabilization, particularly in the CNS. *In vitro work* shows, that when HSP90 is inhibited, there is a notable decrease in the levels and activity of LATS1/2 (Huntoon et al., 2010) and NDR1 (Enomoto et al., 2013). From a mechanistic standpoint, it has been proposed that the HSP90 isoform HSP90β plays a role in inhibiting the proteasomal degradation of LATS mediated by SMURF1 (Qu et al., 2023). Furthermore, the build-up of methylglyoxal (MG) in tumor cells results in increased glycation of HSP90. This glycation, in turn, disrupts the interaction between HSP90 and LATS1, causing impairments in Hippo signaling. Consequently, this disruption is associated with heightened cellular growth and an increased potential for metastasis (Nokin et al., 2016). HSP90 also plays a crucial role in the disassociation and reactivation of LATS2 protein aggregates that form during heat shock. These aggregates, in turn, induce protein-phosphatase-1-dependent dephosphorylation of LATS2, highlighting the indispensable function of HSP90 in the dynamic regulation of LATS2 in response to protein stress (Jiang et al., 2021). It is also worth noting that in yeast, the LATS1/2 homolog CBK1 has been demonstrated to modulate HSP70 and inhibit the nuclear toxicity associated with huntingtin protein aggregates (Wolfe et al., 2014). Mirroring this pro-survival mechanism, HSP70 in mammals can form a complex with BAG3 that regulates the early aggresome formation in response to the accumulation of abnormal polypeptides in a LATS1-dependent manner (Meriin et al., 2018).

In summary, it is evident that NDR kinases play a crucial role in regulating proteostasis, specifically in governing protein stability through interaction with HSP90 or HSP70, and also the eventual protein degradation through autophagy. These kinases contribute to various points in the autophagic pathway, and it is crucial to underscore that their global impact on autophagy is highly complex; depending on the specific level of the pathway at which they participate, the distinct stress conditions inducing autophagy, and the particular cell type involved. Remarkably, their importance is especially evident in neuronal proteostasis, and the disturbance of NDR kinases during aging could potentially signify a previously unrecognized aspect of age-related neurodegenerative diseases. Finally, considering the crucial role of autophagy in memory maintenance during aging (Glatigny et al., 2019), this dysregulation may contribute to cognitive decline and needs to be further explored.

## Mitochondrial dysfunction

For nearly 80 years, researchers have theorized that mitochondria play a critical role in the regulation of lifespan, dating back to the proposal of the mitochondrial theory of aging. Even though it is considered an outdated theory, it is clear that mitochondrial function plays a very important role in the regulation of lifespan and aging, not only by producing free radicals that damage cells over time but by impairing energy metabolism, homeostasis, creating oxidative stress and dysregulating apoptosis (Lima et al., 2022). NDR kinases also seem to have a pivotal role in mitochondrial biology, particularly in mitochondrial quality control (MQC), which is a system that involves the activation of several signaling pathways that ensure mitochondrial homeostasis. One of the main functions of the MQC is the clearance of damaged mitochondria through mitophagy and mitochondrial biogenesis through the transcription of mitochondrial genes. The PTEN-induced kinase 1 (PINK1)/PARKIN signaling pathways is one of the main effectors of MQC. It was shown that in *Drosophila* upon mitochondrial damage by increased ROS production through rotenone administration, Pink1 promotes the localization of Trc to mitochondria by the phosphorylation at the T453 in a Torc2-dependent manner, and by increasing the Trc phosphorylation at S292 via an unidentified signaling pathway (Wu et al., 2013). The mechanism by which the NDR kinases might be involved in mitochondrial clearance was explored further and it was demonstrated that phosphorylated Trc in the mitochondria interacts with Atg1, ortholog of mammalian ULK1/2, the mitochondrial transporter protein Miro and leads to Parkin phosphorylation which promotes the activation of pathways involved in MQC. Moreover, the mammalian ortholog NDR1 also localizes to the outer membrane of mitochondria and *Stk38* knockdown leads to the accumulation of damaged mitochondria due to dysfunctional PINK1/PARKIN pathway (Wu et al., 2013). Another study demonstrated that the mechanism behind the accumulation of damaged mitochondria in *Stk38* KO cells involves PINK1/PARKIN mediated mitophagy and NDR1 deficiency decreases the cell survival of the transformed cells after ECM detachment (Bettoun et al., 2016), hinting that a common maladaptive feature of NDR kinases is tumor metastasis. In neurons, a potential role of NDR1/2 in MQC has been implicated by the finding that murine neurons lacking NDR1/2 display fragmented and rounded mitochondria (Roşianu et al., 2023), a mitochondrial phenotype also observed in neurodegenerative conditions (Su et al., 2010). Interestingly, yeast of the species *Neurospora crassa* with mutations in COT-1 exhibit a higher prevalence of mitochondria with irregular shapes (Gorovits et al., 2000), suggesting an evolutionarily conserved feature of NDR kinases in mitochondrial biology.

Even though NDR kinases have been linked directly to the regulation of MQC through one of the most important pathways involving PINK1/PARKIN, direct evidence is still missing that demonstrates the role of this family of kinases in energy metabolism, oxidative phosphorylation and related processes that are a consequence of mitochondrial activity. Some indirect evidence comes from observations related to the other mammalian NDR kinases LATS1 and LATS2. Cells that are actively dividing rely on glutamine as a metabolic source to support the building of molecules needed for growth and to replenish the carbon pool within the mitochondria. Increased ROS production after glutaminolysis inhibition activates RAS homolog family member A (RhoA), which suppresses LATS1 phosphorylation. This event prevents the phosphorylation of YAP1 resulting in its nuclear transport and transcription of downstream targets such as Sestrin 2, which leads to suppression of mTORC1 and activates survival mechanisms such as autophagy (Kim et al., 2023). Another pathway involves SMAC (second mitochondria-derived activator of caspases), where LATS1 interacts with SMAC and promotes the ubiquitination of apoptosis inhibitor proteins such as X-linked inhibitor of apoptosis (XIAP) (García-Gutiérrez et al., 2022) that participate in mitochondrial permeabilization and cytochrome c release (Zhao et al., 2020).

## Genomic instability

It is commonly accepted that as organisms age, an interplay of the elevated rate of genetic mutations with the decline in DNA repair efficiency leads to genomic instability (Gorbunova et al., 2007). Accumulation of various DNA damage exposures, both from environmental and endogenous factors such as ROS and replication errors, are the main threats to genomic integrity. Particularly, the brain has a very high oxygen demand and is enriched with copper and iron molecules that actively participate in ROS generation, which results in substantial ROS-mediated oxidative stress on the genome (Singh et al., 2019). Neurons are one of the longest-lived cells in the body and have a high metabolic activity, thus, strongly depend on DNA repair mechanisms to sustain proper genomic function (Reid et al., 2021).

NDR kinases have been shown to regulate key processes that are involved in regulating DNA repair pathways. It has been demonstrated that NDR1 can be activated by hydrogen peroxide, an oxidative agent that can trigger DNA damage, and modulate metabolic pathways involved in oxidative stress response (Enomoto et al., 2012). Double-strand breaks (DSBs) induced by mutagenic agents represent the most deleterious DNA damage. Ataxia-telangiectasia mutated (ATM) is one of the main proteins involved in the orchestration of the DDR, along with the ATM-Rad3-related (ATR) kinases and the ubiquitin-like UFMylation pathway (Fang and Pan, 2019). Importantly, ATM kinases are also implicated in the sustained DDR in senescent cells, suggesting their potential as pharmacological targets for mitigating the effects of aging (Zhao et al., 2020). Notably, both NDR1 and NDR2 harbor binding motifs for Ubiquitin-fold modifier 1 (UFM1), the main effector of UFMylation, and research using human cell lines has shown that NDR1 is recruited to DSBs in response to DNA damage (Qin et al., 2020), and that loss of NDR1 strongly sensitizes the DNA to damage induced by ionizing radiation. Furthermore, it was shown that NDR1-mediated ATM activation is crucial for DNA repair (Qin et al., 2020). A parallel study validated this observation and noted that, although primarily localized in the cytoplasm, NDR1 accumulates in the nucleus following UV irradiation and confirmed that silencing of NDR1 diminishes the activity of ATR-mediated DNA repair (Park et al., 2015). It has also been shown that NDR1 promoter activity can be controlled by specificity protein 1 (SP1) (Enomoto et al., 2013), a transcription factor that is degraded by DNA damage-induced ATM activity (Swift and Azizkhan-Clifford, 2022), which might provide the potential feedback mechanism for the repair machinery. Along with ATM and ATR, the DNA-PKc, are the most important mediators of the complex DDR network (Menolfi and Zha, 2002). It should be noted that DNA-PKc plays a pivotal role in the non-homologous-end-joining (NHEJ) repair mechanism which is the primary pathway for repairing DSBs in non-dividing cells such as neurons (Yue et al., 2020). It has been further demonstrated that NHEJ efficiency decreases in both neurons as well as astrocytes during aging and contributes to genomic instability in rats and mice, respectively (Vyjayanti and Rao, 2006; Vaidya et al., 2014). Interestingly, DNA-PKc has been shown to activate NDR1 in human glioblastoma cells (Shiga et al., 2020). Given that NDR2 is the main NDR kinase in the adult brain, we suggest that NDR2 might play a very important role in maintaining genomic stability in aging neurons that has not been explored up to this date. Along this line, a large-scale analysis of the phospho-proteome after the activation of the DNA damage response revealed that the mouse NDR2 kinase is one of the substrates of DNA-damage-induced ATM/ATR activity (Matsuoka et al., 2007).

Another finding implicates NDR2 in the Ribosomal DNA (rDNA) integrity, which as one of the most active parts of the eukaryotic genome, is highly susceptible to damage during aging (Kasselimi et al., 2022). RASSF1A is one of the main mediators of rDNA repair that is recruited to rDNA breaks and mediates ATM signaling (Tsaridou et al., 2022). Interestingly, it is known that in the context of DDR, RASSF1A recruits LATS1 (Pefani et al., 2014), and furthermore, RASSF1A can interact and inhibit NDR2 in transformed cells (Keller et al., 2019). NHEJ is also crucial to maintain efficient neurogenesis throughout the lifespan and to ensure the seamless integration of adult-born neurons into the circuitry. Previous studies have shown that neurogenesis decreases notably during aging and this impairment is well-linked to the aging-associated cognitive decline (Lupo et al., 2019; Navarro Negredo et al., 2020). Cell cycle checkpoints are crucial for controlling genomic stability during cell division since they ensure the accuracy of the genome and can trigger DNA repair mechanisms in case of genomic instability. As established before, NDR kinases serve critical roles in the cell cycle progression and the physical segregation of chromosomes after replication as a key factor for the cell cycle and genomic integrity. In this context, it is known that human NDR2 is translocated to the centrosomes in mitosis progression and modulating NDR2 expression results in over or under-duplication of centrosomes (Hergovich et al., 2007). *Lats1* KO mice display increased centrosome overduplication, chromosomal misalignment and deficiency in cytokinesis (Yabuta et al., 2013). NDR1 is also implicated in the mitotic spindle formation and its activity is strictly regulated during kinetochore-microtubule interactions (Yan et al., 2015). Lastly, it has been reported that LATS can inhibit MDM2, which is required for p53 regulation in chromosome number maintenance during mitosis (Aylon et al., 2006).

Overall, the NDR kinases seem to be very important contributors to genome stability and are deeply connected to the DDR network mainly by ATM and DNAPKc-dependent pathways. They also participate in genomic stability by regulating the cell cycle, and more directly, in the segregation of the chromosomes. In the context of neuronal aging, cognitive decline in the human brain is notably associated with a significant downregulation of genes related to learning, memory and synaptic plasticity as well as an increase in DNA damage and corresponding reduction of repair mechanisms (Lu et al., 2004). Taken together, further exploration of the NDR kinases in the maintenance of genomic stability with a particular focus on DDR in neuronal aging is a compelling avenue for future research.

## Epigenetic alterations

One of the consequences of aging on the genome is loss of epigenetic information over the lifespan due to several mechanisms that include alterations in chromatin remodeling, post-translational modification of histones like H3K56ac, H4K16ac, H3K4me3, H3K9me3, and H3K27me3, DNA methylation patterns and regulation of non-coding RNAs (ncRNAs) across different species (Yang et al., 2023). The exact roles of NDR kinases in epigenetic remodeling are still scarce, however, it was shown that In HeLa Cells LATS2 binds to the Polycomb Repressive Complex 2 (PRC2) and increases its histone-methyltransferase activity through phosphorylation, causing an increase in H3K27me3 (Torigata et al., 2016), emphasizing the role of NDR kinases in the control of the epigenetic architecture. H3K27me3 is a modification that participates in gene silencing and interestingly, age-related decrease of H3K27me3 is regarded as one of the main age-related features of histone modification and has been observed in several animal models and yeast (Wang et al., 2022). Moreover, cells taken from Hutchinson-Gilford Progeria Syndrome (HGPS) patients, characterized by rapid aging, display a similar H3K27me3 decrease (Shumaker et al., 2006). Although an alteration in LATS2-PRC2 interaction during aging has not been experimentally confirmed, this evidence hints that further studies are necessary to explore the role of LATS2 in histone methylation in aging. On the other hand, increased H3K27me3 causes a loss of function in mammalian mesenchymal stem cells and muscle satellite cells during aging (Noer et al., 2009) and has been linked to aging in killifish and mouse brain (Baumgart et al., 2014). Overall, this suggests that the effect of LATS2 through histone-methylation could be complex, species and tissue-specific but might accelerate aging in neuronal tissue.

## Altered intracellular communication

The complex function of the nervous system heavily depends on communication between neurons, collaborative support provided by non-neuronal cells and the interaction of neurons and glia with their extracellular environment. During aging, significant alterations of these intercellular communication pathways have been demonstrated, ranging from an aberrant secretion of inflammatory response (Ransohoff, 2016) to alterations in the mechanical properties of the brain (Elkin et al., 2010) such as increased stiffening. There is evidence that hints that as important mediators of the immune response and central players in the mechanosensing via the Hippo pathway, NDR kinases may have maladaptive features that impair brain function during aging.

TNF-α is an upstream ligand of NF-κB signaling for the inflammatory response and has been shown to accumulate during aging (Bruunsgaard, 1999). It has been reported that the deficiency of NDR1 inhibits TNF-α-mediated transcriptional responses (Ma et al., 2017). Notably, TNF-α can activate both NDR1 and NDR2, and silencing of *Stk38* and *Stk38l* significantly reduces TNF-α-mediated cellular effects, including apoptosis (Vichalkovski et al., 2008). Interestingly, *Stk38* KO mice display increased TNF-α and interleukin production, which suggests that NDR kinases are also a limiting factor for inflammation response (Wen et al., 2015). In addition to TNF-α, several other interleukins, such as Interleukin-17 (IL-17) whose receptors exhibit a high expression in the brain (Das Sarma et al., 2009), show a significant increase in the aging murine brain (Porcher et al., 2021). Conversely, while NDR1 facilitates IL-17 signaling by disinhibiting the IL-17 receptors (Ma et al., 2017); NDR2 has been shown to block the IL-17 pathway and silencing of NDR2 enhances IL-17-induced inflammatory response (Vichalkovski et al., 2008). The brain is highly susceptible to blood-circulating cytokines such as Interferon-1 (IFN1), particularly at the choroid plexus which serves as an interface between the periphery and central nervous system. Studies have shown that blocking the exaggerated IFN1 response in aged mouse brains can potentially restore cognitive impairments and neurogenesis defects associated with aging (Baruch et al., 2014). Regarding the other mammalian kinases, LATS1 is a previously recognized important player in the IFN1 response. LATS1 is recruited to IFN1 receptors, undergoes rapid activation upon IFN1 binding, and plays a pivotal role in mediating downstream transcriptional signaling (Zuo et al., 2022). Besides cell-to-cell inflammatory communication, alterations in growth signal transductions such as IGF (Wrigley et al., 2017) and vascular endothelial growth factor (VEGF) (Grunewald et al., 2021) are commonly observed in aging organisms. A study demonstrated that NDR2 is activated following IGF stimulation and that a hyperactive NDR2 mutant can initiate downstream cell survival pathways even in the absence of the IGF ligand (Suzuki et al., 2006). Moreover, it has been reported that in multiple cell lines, LATS1/2 kinase activity is inhibited by VEGF signaling and the PI3K pathway, which is required for the VEGF effects on the modulation of the Hippo pathway (Azad et al., 2018).

### NDR kinases control extracellular matrix communication

Several studies have reported that the stiffness of the brain tissue changes throughout aging (Gefen et al., 2003; Sack et al., 2009; Elkin et al., 2010). This altered mechanical signaling from the extracellular space can be sensed via integrin receptors on the membrane and Hippo pathway and consequently triggers cytoskeleton remodeling as a response (Cai et al., 2021). Increased stiffness of brain tissue may cause age-related alterations such as loss of function of progenitor cells over time (Segel et al., 2019) or modulation of neuronal morphology (Si et al., 2023). The relationship between NDR kinases and ECM is tightly conserved across species. The LATS kinase homolog DBF2 in *S. cerevisiae* phosphorylates and activates both chitin synthase CHS2 and CYK3 during cell division. The localization of CYK3 is dependent on DBF2, setting up a mechanism for the direct control of the primary septum remodeling during the cell cycle, which is equivalent to the metazoan ECM (Oh et al., 2012). NDR kinases contribute to these processes both through the Hippo pathway and as critical regulators of integrin-mediated intracellular signaling. In this line, we have previously shown that NDR2 can modulate integrin receptor trafficking and activity in T cells (Waldt et al., 2018) and murine neurons (Rehberg et al., 2014). Furthermore, NDR kinases regulate the arborization of dendrites and axons as well as spine development and synaptic function in mammalian neurons (Ultanir et al., 2012; Rehberg et al., 2014). We showed that NDR2 controls substrate selectivity by regulating integrin subunit availability in growth cones during neurite growth (Demiray et al., 2018). Notably, integrin receptor regulation is also addressed in aging-associated pathologies, as enhancing integrin signaling holds promise in alleviating impairments in blood-brain barrier integrity in rats (Halder et al., 2023) and promoting cellular regeneration (Rozo et al., 2016; Ojha et al., 2022).

Moreover, functioning as a scaffold, the ECM supports cells, allowing them to perceive external forces and maintain their shape (Hynes, 2009) by transducing mechanical cues from the environment to the cells (Humphrey et al., 2014). Along this line, previous studies revealed a functional link between the ECM mechanotransducer glycoprotein AGRIN and Hippo pathway mediator YAP. AGRIN, as a sensor of ECM stiffness, increases the stability of YAP by the focal adhesion and LRP4/MUSK receptor pathways. AGRIN inhibits the focal adhesion assembly of Hippo pathway proteins by promoting ILK-PAK1 signaling and decreasing MERLIN and LATS1/2 interaction (Chakraborty et al., 2017). AGRIN also plays a role in supporting murine adult hippocampal neurogenesis (Zhang et al., 2019), facilitating synaptogenesis in developmental stages in a rat model of post-exercise stroke (Zhang et al., 2020), maintaining murine adult NMJs (Samuel et al., 2012), and is implicated in the pathogenesis of AD (Donahue et al., 1999; Verbeek et al., 1999). Altogether, NDR kinases could potentially be involved in AGRIN function of the developing and aging brain.

Beyond its mechanical function and role in transduction, ECM serves as a cohesive substrate for cell movement. This adhesive property is critical during cell migration and in processes such as development, wound healing, and regeneration (Rolfe and Grobbelaar, 2012; Kular et al., 2014). Fibronectin (FN), an important member of brain ECM, has a neuroprotective function in axonal regeneration and neurite outgrowth of cortical and hippocampal adult mice neurons (Tonge et al., 2012) and it diminishes during aging in the brain (Wang et al., 2011). Interestingly, it has been shown that FN adhesion increases the accumulation of YAP in the nucleus. Mechanistically, FN activates the focal adhesion kinase (FAK), which negatively regulates LATS1/2 via PI3K signaling. Reduction of LATS1/2 activity leads to YAP nuclear accumulation and transcriptional response in response to FN adhesion (Kim and Gumbiner, 2015). Notably, integrin receptors on the membrane recognize the FN in ECM and their activity can be modulated by NDR2 kinase activity (Rehberg et al., 2014), indicating the potential role of NDR Kinases in the neuroprotective functions of FN. Furthermore, cytoskeleton remodeling is a key downstream target of ECM signals and alterations in cytoskeletal dynamics have been closely associated with aging (Starodubtseva, 2011; Zahn et al., 2011; Lai and Wong, 2020). Along this line, *in vitro* experiments using human cell lines have shown that disturbance of microtubules with nocodazole reduces the activity of LATS1/2, while the disruption of the actin cytoskeleton with latrunculin B activates LATS1/2 (Zhao et al., 2012), implying the involvement of the cytoskeleton in the LATS-dependent regulation of YAP activity.

Taken together, the modulation of immune signaling between cells, regulation of neuronal shape and synaptic signaling, and the transduction of mechanosignalling driven by the extracellular matrix underscore the crucial role of NDR kinases in governing intercellular communication within the brain. However, further studies are necessary to specifically address the role of NDR kinases in the context of age-associated alterations in cellular communication in the nervous system.

## Closing remarks

**Figure 1:**
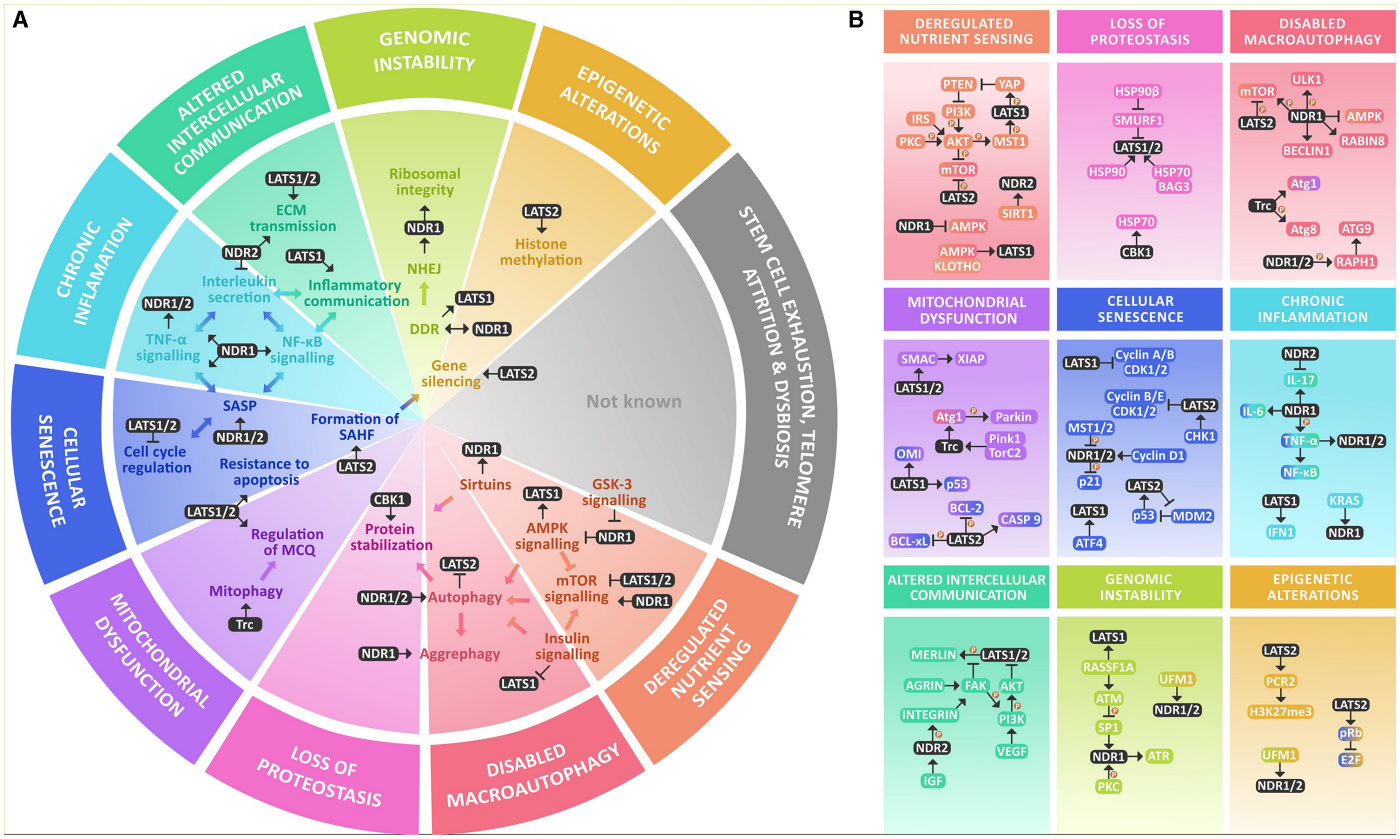
NDR kinases within the hallmarks of aging at a glance. **(A)** NDR kinases are involved in various key processes that are altered within the hallmarks of aging. They are known to regulate AMPK and mTOR signaling and are modulated by GSK-3 and Sirtuins. NDR1/2 kinases play a crucial role in regulating autophagy and integrating growth signals from AMPK, mTOR, and insulin signaling. They also participate in protein stabilization through chaperones. In mitochondrial dysfunction, NDR kinases participate in mitophagy and MQC. They have a significant role in inflammation and cellular senescence by participating in the formation of SAHF, regulating the cell cycle, resistance to apoptosis, and increasing inflammation through SASP, TNF-α, NF-κB, and interleukin secretion. NDR kinases also participate in intercellular communication by modulating ECM transmission and regulating inflammatory communication, in genomic instability mainly through NHEJ and DDR, and in epigenetic alterations by gene silencing through histone methylation. However, their roles in stem cell exhaustion, telomere attrition, or dysbiosis are not yet reported. **(B)** NDR kinases participate in nutrient signaling through a complex interplay between the major nutrient sensing pathways: AMPK, mTOR & Insulin signaling, and though the functional outcome is not known yet, they are also deacetylated by SIRT1. For their role in the loss of proteostasis, they participate in protein stability by regulating molecular chaperones like HSP70 and are in turn stabilized by it as well as HSP90. NDR kinases are regulators of macroautophagy (mTOR, ULK1, AMPK, BECLIN1, Atg1, Atg8, and Atg3). Within mitochondrial dysfunction, they are known regulators of MQC and mitophagy by a Pink1/Parkin-dependent mechanism that involves Atg1. NDR kinases participate in cellular senescence by regulating the cell cycle, mainly regulating Cyclin A/B or Cyclin B/E and CDK protein complexes and modulation of E2F through pRB. Additionally, they regulate p21 and p53 signaling. LATS1 is also a downstream target of ATF4. They also provide resistance to apoptosis through the same mechanism and interact with BCL-2, BCL-xL, and BAX in parallel with SMAC and XIAP. They might be involved in regulating SASP and Chronic inflammation, mainly by increasing TNF-α and NF-κB activation and proinflammatory interleukin secretion, mainly IL-6, as well as IFN1For altered intercellular communication, they participate as signal transducers of growth factors like VEGF and IGF. They participate in inflammatory communication through TNF-α, NF-κB, and IL-17 and regulate ECM signaling through activation of the integrins. They might participate in genomic instability by regulating DDR and rDNA integrity by coordinating the response of ATM, ATR, and DNA PKc. Finally, there is evidence that links NDR kinases to the regulation of the epigenome by increasing the methylation of the Polycomb repressive complex 2.

There is abundant evidence supporting the crucial involvement of NDR-Kinases in diverse cellular processes underlying aging. This review summarizes their key roles in a comprehensive way so that it reflects the hallmarks of aging, particularly in cellular senescence, chronic inflammation, deregulated nutrient sensing, loss of proteostasis, impaired macroautophagy, and to a lesser extent, altered intracellular communication, mitochondrial dysfunction, genomic instability, and epigenetic alterations, with an increased focus on neuronal biology (Figure 1).

In summary, NDR kinases seem to be key components of the complex changes observed in senescent cells. They might contribute to the arrest of proliferation, chronic inflammation through the regulation of constituents of the SASP such as TNF-α, IL-6, and NF-κB, and the resistance of senescent cells to apoptosis. Interestingly, NDR1/2 and LATS1/2 often seem to have opposed roles in these processes which shows that understanding the molecular mechanisms that maintain a balance between their activity is a promising target to understand the nuances of the regulatory network in senescent cells. Another avenue that still needs further exploration is their involvement in neuronal senescence, which has only recently gained recognition as a feature of aging neurons. Another consequence is that by modulation of interleukins, IGF, VGEF, integrin signaling or mechanosensing through the ECM, which is sensitive to ECM proteases present in the SASP, NDR kinases could participate in altered intercellular communication during aging. Besides, it is plausible to assume that NDR-Kinases play an important role in age-dependent stem cell exhaustion as essential regulators of cell cycle progression and previously shown players in stem cell function (Mo et al., 2014).

In the context of nutrient sensing, NDR Kinases play an important role in insulin, mTOR, and AMPK signaling. These pathways, including SIRT1, have been demonstrated to be the central metabolic pathways that dictate lifespan and the rate of aging across all eukaryotes. While NDR kinases have a complex intercommunication with mTOR and AMPK signaling that can result in both inhibition or activation of the pathways; increased insulin signaling often leads to activation of NDR kinases. There is a substantial body of evidence that indicates NDR kinases as master regulators of autophagy, supported by their involvement in macroautophagy, CASA, and mitophagy. NDR kinases actively contribute to DNA repair through the orchestration of the DDR and NHEJ in neurons. Together with their functions in chromosomal alignment and maintenance, NDR kinases demonstrate their importance as components of the cellular machinery that maintains genomic stability under stress and replication. There is also some evidence that points out their role in epigenetic regulation, particularly by increasing H3K27me through the methyltransferase activity of PRC2. Although there is no evidence indicating that NDR kinases play a role in the maintenance of telomeres, given their close involvement in DNA biology, the possibility of their involvement cannot be entirely dismissed and warrants further attention. One last hallmark of aging that remains to be addressed is dysbiosis. The paracrine effect that the microbiome exerts over other cells has been explored only in recent years as a mechanism that regulates lifespan and aging, and it isn't surprising that there is no evidence linking NDR kinases to dysbiosis, given there are no prokaryotic analogs of the NDR kinases. The mechanism by which the microbiome regulates lifespan remains poorly understood, therefore an intriguing area of exploration relates to the signals originating from the microbiome and the mechanisms of how cells perceive them. It is known that the primary receptors for microbiome signals are the immune system and the CNS (Zheng et al., 2020; Park and Kim, 2021), both of which have an active participation of NDR kinases. These observations raise the possibility that NDR kinases might participate in the transduction of gut microbiota alterations during aging.

It is evident that NDR kinases have an intricate connection to many of the cellular and molecular processes that are considered to be the cause of aging by our current understanding. The functional outcomes of many of the interactions described in this review are not always clear, with the existence of much contradictory evidence. For instance, while NDR kinases are required for proper cell function, they also participate in cancer and disease development. They promote cell cycle progression, but in some contexts, they also induce apoptosis and cellular arrest. NDR kinases both seem to activate and inhibit central metabolic cascades or promote and downregulate inflammatory signals. As a final remark, we want to propose that many of the observed contradictions in the literature regarding the function of NDR kinases arise from the fact that they might have evolved having antagonistic pleiotropic functions in aging. The current paradigm of the evolution of aging suggests that aging occurs by the accumulation by natural selection of genes that have antagonistic pleiotropic features that increase fitness during a young age, but that also show maladaptive features during aging, favoring a trade-off between reproduction and lifespan (Chistyakov and Denisenko, 2019). The evidence presented so far indicates that NDR kinases display these classic antagonistic pleiotropic functions that contribute to the loss of fitness during aging. If they are studied within this new perspective, many of the opposite roles that they exhibit can be easily understood, paving the way for further comprehending the interconnectivity that exists among the hallmarks of aging and given their importance in neuronal biology, also understanding more about the mechanisms that lead to age-related loss of cognitive function.

## Author contributions

KJ: Data curation, Investigation, Writing – original draft, Conceptualization. MA: Conceptualization, Data curation, Investigation, Supervision, Writing – original draft, Writing – review & editing. YD: Investigation, Writing – original draft, Data curation, Writing – review & editing. AL: Investigation, Writing – original draft. OS: Funding acquisition, Project administration, Resources, Supervision, Validation, Writing – review & editing.

## Glossary

AD, Alzheimer's Disease; AKT/PKB, Protein Kinases B; ALS, Amyotrophic Lateral Sclerosis; AMOT, Angiomotin; AMOTL, Angiomotin-like Protein; AMPK, Amp-activated Protein Kinase; ATF4, Activating Transcription Factor 4; ATG, Autophagy-related Protein; ATM, Ataxia-Telangiectasia Mutated; ATR, ATM-and Rad3-Related; BAG3, BCL-2-associated Athanogene 3; BC, Breast Cancer; BCL-2, B-cell Lymphoma 2; BCL-xL, B-cell Lymphoma-extra Large; CASA, Chaperone-assisted Selective Autophagy; CBK1, Cell Wall Biosynthesis Kinase; CBP, Creb-binding Protein; CDC, Cell Division Control Protein; CDK, Cyclin-dependent-kinase; CDKN1A, Cyclin-dependent-kinase-inhibitor 1a; CHEK1, Checkpoint Kinase 1; DDB1, DNA Damage-binding Protein 1; DDR, DNA Damage Response; DNA-PKcs, DNA-dependent Protein Kinase, Catalytic Subunit; DREAM, Dimerization Partner, Rb-like, E2f and Multi-vulval Class B-complex; DSBs, Double Stranded Breaks; ECM, Extracellular Matrix; ERK, Extracellular-signal Regulated Kinases; F-actin, Filamentous Actin; FAK, Focal adhesion kinase 1; FOXO, Forkhead Box O; GATA, Gata Binding Protein 4; GSK-3β, Glycogen Synthase Kinase 3 Beta; HFD, High Fat Diet; HGPS, Hutchinson-Gilford Progeria Syndrome; HSP, Heat Shock Protein; IFN1, Interferon type-I; IGF1, Insulin-like Growth Factor 1; IIS, Insulin and IGF1 Signaling; IL-6, Interleukin 6; IRS, Insulin Receptor-substrates; LATS, Large Tumor Suppressor; LC3, Microtubule-associated Proteins 1a/1b Light Chain 3; LKB-1, Liver Kinase B1; lncRNAs, Long Non-coding RNAs; MDM2, Mouse Double Minute 2 Homolog; MEKK2, Mitogen-activated Protein Kinase 2; MG, Methylglyoxal; MOB, MPS1 Binder; MQC, Mitochondrial Quality Control; MST, Mammalian Sterile 20-like; mTOR, Mechanistic Target of Rapamycin; mTORC1/2, Mechanistic Target of Rapamycin Complex 1/2; mTiRS130, Mammalian Trappii-specific Subunit 130; Mys, Myospheroid; NAFLD, Nonalcoholic Fatty Liver Diseases; NDR, Nuclear Dbf2-related; NF-κB, Nuclear Factor Kappa B; NHEJ, Non-homologous end joining; NMJ, Neuromuscular Junction; OGT, O-linked N-acetylglucosamine Transferase; OIS, Oncogene-induced Senescence; PD, Parkinson's Disease; PI3K, Phosphoinositide 3-kinase; PINK1, PTEN-induced Kinase 1; pRB, Retinoblastoma Protein; PRC2, Polycomb Repressive Complex 2; PTEN, Phosphatase and Tensin Homolog; PUMA, p53 Upregulated Modulator of Apoptosis; rDNA, Ribosomal DNA; RhoA, RAS Homolog Family Member A; ROS, Reactive Oxygen Species; SAHF, Senescence-associated Histone Foci; SASP, Senescence-associated Secretory Phenotype; SIRT, Sirtuin; SMAC, Second Mitochondria-Derived Activator of Caspases; SMURF1, Smad Ubiquitination Regulatory Factor 1; SOD1, Superoxide Dismutase 1; SP1, Specificity protein 1; TAG, Triacylglycerols; TAZ, Transcriptional Co-activator With Pdz-binding Motif; TEAD, Transcriptionally-enhanced Associate Domain; TNF-α, Tumor Necrosis Factor-alpha; Trc, Tricornered; TRIM27, Tripartite Motif Containing 27; UFM1, Ubiquitin-Fold Modifier 1; ULK1, Unc-51-like Kinase 1; VEGF, Vascular Endothelial Growth Factor; WASP, Wiskott-Aldrich Syndrome Protein; Wts/WTS, Warts; XIAP, X-linked inhibitor of Apoptosis Protein; YAP, Yes-associated Protein; Yki, Yorkie.
